# Supplementary material for: FUS (fused in sarcoma) is a component of the cellular response to topoisomerase I–induced DNA breakage and transcriptional stress
Source: Life Sci Alliance. 2019 Feb 26;2(2):e201800222. doi: 10.26508/lsa.201800222 (PMC6391683; doi:10.26508/lsa.201800222)
Supplement: Supplementary file 1 [file LSA-2018-00222_TableS1.docx]

**Table S1.**

| **Primer** | **Sequence** | **Use** |
| --- | --- | --- |
| FOSEX1 fw | 5’-ACTACCACTCACCCGCAGAC-3’ | qRT-PCR |
| FOSEx2 rv | 5’-TGGTCGAGATGGCAGTGAC-3’ | qRT-PCR |
| FOS Int1 fv | 5’-GGCTTTCCCCTTCTGTTTTG-3’ | qRT-PCR |
| FOS Int2 rv | 5’-AGATCCTCAGCAAGAGAAC-3’ | qRT-PCR |
| FOS Ex4 fw | 5’-CTTCCCTTGATCTGACTGG-3’ | qRT-PCR and ChIP-qPCR |
| FOSEx4 rv | 5’-CTGATGCTCTTGACAGGTTC-3’ | qRT-PCR and ChIP-qPCR |
| FOSpro fw | 5’-AGGTTTCCACGGCCTTTCC-3’ | ChIP-qPCR |
| FOSpro rv | 5’-TTTCGCAGTTCCTGTCTCAGAG-3’ | ChIP-qPCR |
| rDNAPro fw | 5’-GCCCCGGGGGAGGTAT-3’ | ChIP-qPCR |
| rDNAPro rv | 5’-GAGGACAGCGTGTCAGC-3’ | ChIP-qPCR |
| 18S fw | 5’-GTTGAACCCCATTCGTGATG-3’ | ChIP-qPCR |
| 18S rv | 5’-GGGACTTAATCAACGCAAGC-3’ | ChIP-qPCR |
| ITS1 fw | 5’-TGTGAAACCTTCCGACCCC-3’ | ChIP-qPCR |
| ITS1 rv | 5'-GGGGTTGCCTCAGGCC-3' | ChIP-qPCR |
| ACT fw | 5’- GAAGTGTGACGTGGACATCC-3’ | qRT-PCR and ChIP-qPCR |
| ACT rv | 5’-CTCGTCATACTCCTGCTTGC-3’ | qRT-PCR and ChIP-qPCR |
| NT ChrV fw | 5’-CTGTACCTGGGGTTCATTCATT-3’ | ChIP-qPCR |
| NT ChrV rv | 5’ CAGTAAGCCGTTCACTCTCAC-3’ | ChIP-qPCR |
